# Supplementary material for: Discrimination and Identification of Aroma Profiles and Characterized Odorants in Citrus Blend Black Tea with Different Citrus Species
Source: Molecules. 2020 Sep 14;25(18):4208. doi: 10.3390/molecules25184208 (PMC7570765; doi:10.3390/molecules25184208)
Supplement: Supplementary file 1 [file molecules-25-04208-s001.pdf]

Supplementary Materials:

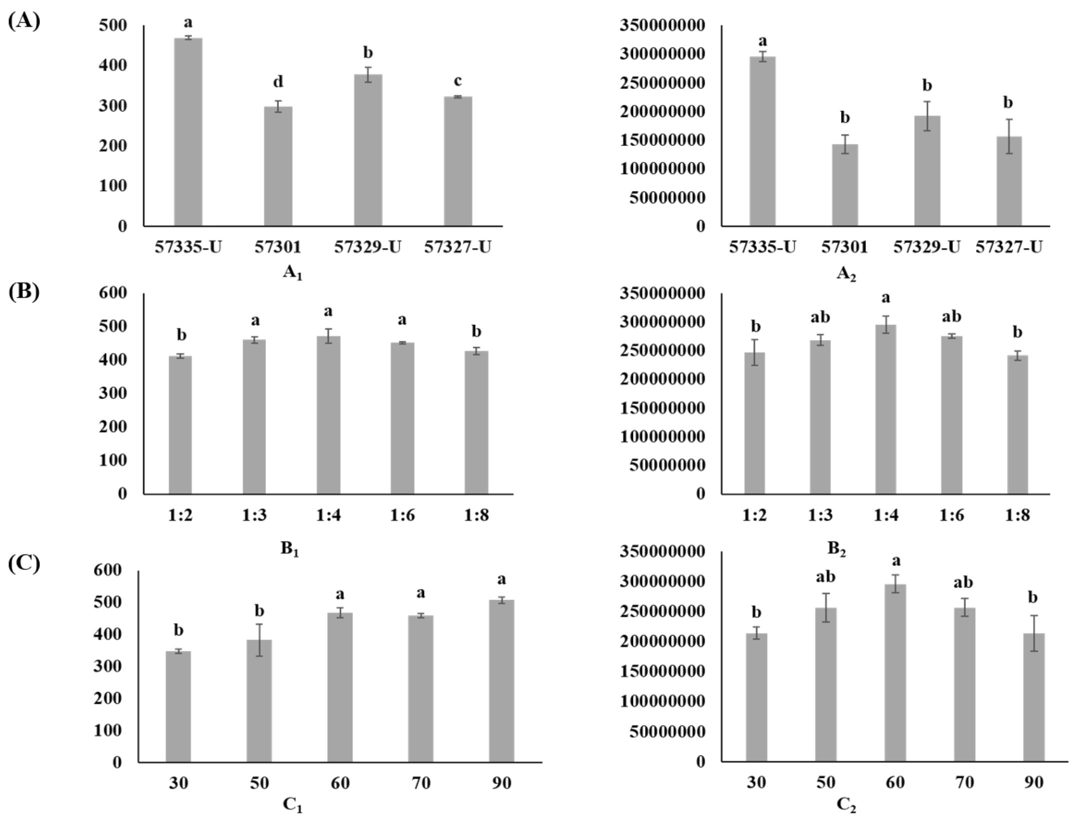

Figure S1. Optimization of citrus blend black tea volatile extraction **(A)**, tea to water ratio **(B)**, extraction temperature **(C)**. **A**$_1$: Total amount of compounds obtained by different types of fiber; **A**$_2$: Total peak area of compounds obtained by different types of fiber. **B**$_1$: Total amount of compounds obtained by different tea to water ratios; **B**$_2$: Total peak area of compounds obtained by different tea to water rations. **C**$_1$: Total amount of compounds obtained by different extraction temperatures; **C**$_2$: Total peak area of compounds obtained by different extraction temperatures.

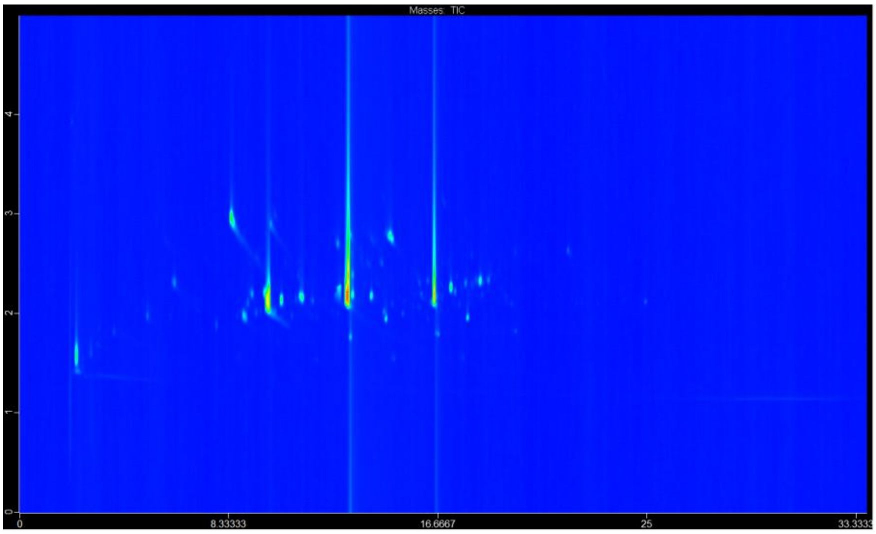

Figure S2. The GC×GC chromatograms of LB-MGHC aroma extracts in Citrus group.

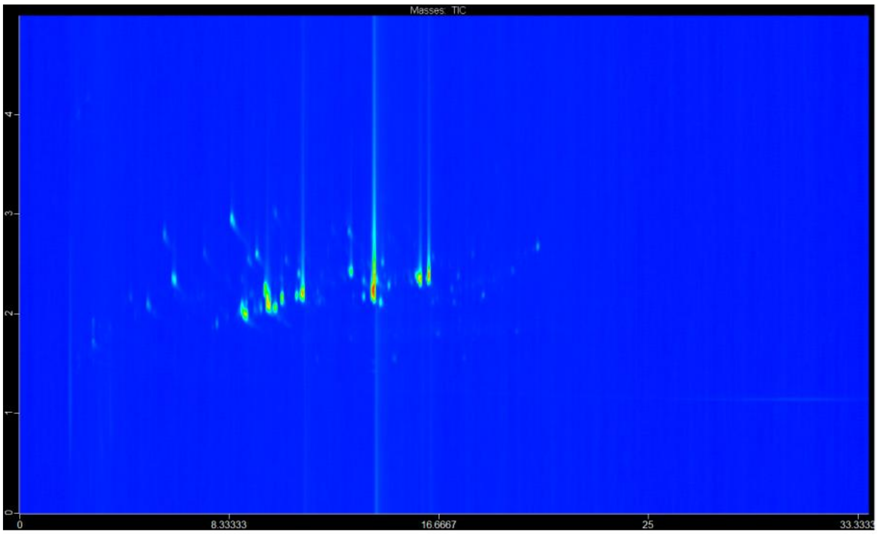

Figure S3. The GC×GC chromatograms of CN-BJHC aroma extracts in Bergamot group.

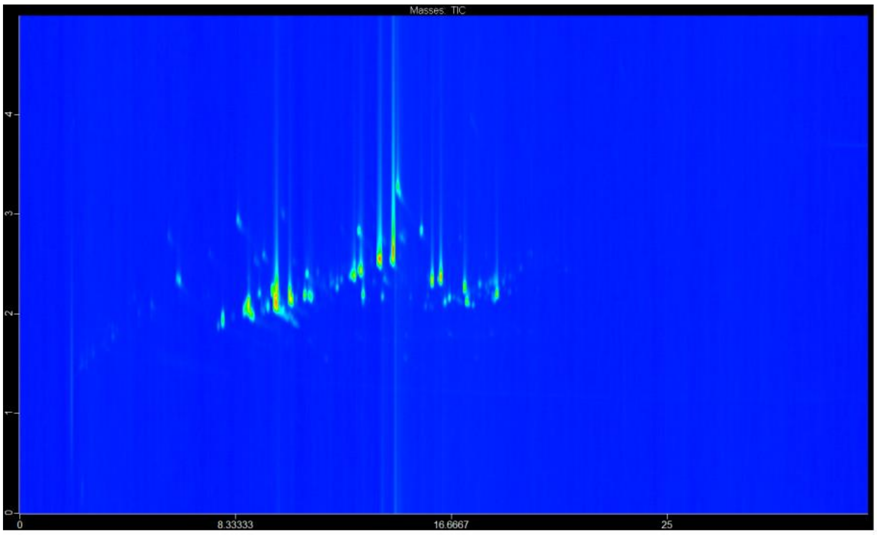

Figure S4. The GC×GC chromatograms of LD-NMHC aroma extracts in Lemon group.

Table S1. Sensory evaluation of 50 citrus blend black tea samples.

Group	No.	Score	Description
Citrus group	1	4.90	Slightly tea flavor, citrus-like, fruity, sweet, floral
	2	4.00	Slightly tea flavor, citrus-like, sweet, floral, fruity
	3	3.02	No tea flavor, sweet, fruity
	4	2.79	Slightly tea flavor, medical
	5	2.89	Slightly tea flavor, floral, medical
	6	3.12	No tea flavor, lemon-like, fruity
	7	3.47	No tea flavor, sweet, citrus-like
	8	3.26	Slightly tea flavor, smokey, medical
	9	2.98	No tea flavor, floral
	10	3.10	Slightly tea flavor, smokey, roasted
	11	3.50	Slightly tea flavor, floral, sweet
	12	2.00	No tea flavor, citrus-like, roasted
	13	3.09	Tea flavor, floral, sweet
	14	4.20	Slightly tea flavor, cinnamon-like, citrus-like, fruity, floral, herbal medical
	15	3.50	Tea flavor, heavy smokey
Bergamot group	16	4.10	Slightly tea flavor, sweet, citrus-like, bergamot-like
	17	1.76	Heavy sour, medical, floral
	18	2.10	No tea flavor, medical
	19	3.50	Heavy medical, wood
	20	1.30	No tea flavor, medical, wood, sweet
	21	4.10	Slightly tea flavor, bergamot-like, jasmine like, floral, fresh, grassy
	22	3.46	Tea flavor, heavy smokey, fruity
	23	2.64	Slight tea flavor, floral,
	24	4.14	Slightly tea flavor, lavender-like, bergamot-like, medical
	25	3.99	Tea flavor, roasted, lemon-like
	26	3.80	Slightly tea flavor, lavender-like, roasted
	27	3.79	Slightly tea flavor, medical, sweet, bergamot-like
	28	4.00	Slightly tea flavor, wood, medical, bergamot-like
	29	3.95	Slightly tea flavor, roasted, floral, herbal
	30	1.80	No tea flavor, medical, sour
	31	2.46	Slight tea flavor, roasted, medical
	32	3.16	Slight tea flavor, medical
	33	1.80	No tea flavor, sour
	34	4.44	Slightly tea flavor, bergamot-like, slightly medical
Lemon group	35	4.10	Have tea flavor, sour lemon-like, fresh
	36	3.20	Slight tea favor, roasted, medical, fresh
	37	2.90	Slight tea favor, medical, sour
	38	3.03	Slight tea flavor, heavy roasted

39	3.46	Tea flavor, medical, wood, sour
40	4.40	Heavy tea flavor, fresh, lemon-like, roasted, sweet
41	4.04	Have tea flavor, fresh, lemon-like, caramel-like
42	3.96	Tea flavor, lemon-like, medical, roasted
43	3.41	No tea flavor, lemon-like, roasted
44	3.68	Slight tea flavor, lemon-like, roasted, wood
45	2.99	Tea flavor, medical, caramel-like, sour
46	3.65	Tea flavor, sweet, floral,
47	4.40	Heavy tea flavor, fresh, lemon-like, flavor, fruity, roasted, sweet
48	3.76	Slightly tea flavor, medical, sweet
49	3.24	Tea flavor, roasted, wood
50	2.00	No tea flavor, sour, roasted

Table S2. Identified volatile compounds and contents in citrus blend black teas [1]

No.	Compounds	CAS number	1st RT (min)	2nd RT (s)	RI	Average relative content (‰) [2]			VIP [3]	*P* Value [4]
						Citrus group	Bergamot group	Lemon group		
	**Esters**									
1	Propanoic acid ethyl ester	105-37-3	3.75	1.82	733.55	2.18±2.18	0.04±0.08	nd	0.7763	0.2
2	n-Propyl acetate	109-60-4	3.75	1.84	733.56	0.03±0.08	0.59±0.52	3.01±4.6	0.7998	0.229
3	(E)-2-Butenoic acid methyl ester	623-43-8	4.5	2.10	771.26	nd	nd	0.03±0.06	0	-
4	Ethyl butyrate	105-54-4	5.08	2.02	800.34	15.98±18.28	0.04±0.08	0.04±0.14	1.1777	0.006
5	Acetic acid butyl ester	123-86-4	5.33	2.03	812.82	0.13±0.2	0.29±0.32	1.51±2.8	1.3096	0.028
6	Formic acid pentyl ester	638-49-3	5.67	1.98	825.31	nd	0.1±0.12	0.06±0.12	0.162	0.064
7	1-Butanol-3-methyl-acetate	123-92-2	6.58	2.03	875.3	0.11±0.16	0.02±0.03	nd	0.1916	0.05
8	2-Propenoic acid butyl ester	141-32-2	7.00	2.13	896.21	nd	0.04±0.07	0.31±0.5	0.2044	0.668
9	(Z)-2-Penten-1-ol acetate	42125-10-0	7.25	2.20	908.41	nd	1.42±0.93	0.58±0.38	0.5545	0.049
10	Acetic acid pentyl ester	628-63-7	7.33	2.08	912.32	nd	0.13±0.09	0.04±0.04	0.1905	0.01
11	Butyrolactone	96-48-0	7.42	3.73	921.6	nd	0.1±0.11	0.29±0.31	0.1639	0.082
12	(Z)-3-Hexen-1-ol formate	33467-73-1	7.50	2.16	920.37	nd	nd	0.06±0.1	0.1743	0.037
13	Hexanoic acid methyl ester	106-70-7	7.58	2.10	924.32	0.03±0.04	0.08±0.05	0.49±0.53	0.2269	0.516
14	(E)-3-Hexenoic acid methyl ester	13894-61-6	7.67	2.22	928.44	nd	0.02±0.02	0.05±0.05	0.0743	0.098
15	Hexanoic acid ethyl ester	123-66-0	9.08	2.11	996.33	6.57±8.27	0.01±0.01	0.04±0.05	1.1049	0.204
16	Acetic acid hexyl ester	142-92-7	9.42	2.12	1008.4	1.26±1.86	0.91±0.95	1.45±2.62	0.4477	0.791
17	4-Hexanolide	695-06-7	10.33	3.33	1057.3	0.01±0.02	0.02±0.03	0.07±0.07	0.0767	0.348
18	Formic acid phenylmethyl ester	104-57-4	10.83	2.85	1080.9	0.01±0.01	0.02±0.04	0.02±0.04	0.0654	0.464
19	Heptanoic acid ethyl ester	106-30-9	11.08	2.11	1092.3	0.24±0.19	nd	nd	0.0061	-
20	Benzoic acid methyl ester	93-58-3	11.17	2.81	1096.9	0.13±0.07	0.11±0.04	0.33±0.22	0.1402	0.057

21	Octanoic acid methyl ester	111-11-5	11.67	2.13	1122.1	1.45±2.17	0.2±0.16	0.13±0.15	0.652	0.08
22	Benzyl acetate	140-11-4	12.50	2.82	1161.8	0.11±0.15	26.46±44.53	0.21±0.09	2.3411	0
23	Benzoic acid ethyl ester	93-89-0	12.58	2.87	1174.7	5.32±5.41	0.07±0.05	0.21±0.26	1.1631	0.101
24	(Z)-Butanoic acid, 3-hexenyl ester	16491-36-4	12.83	2.20	1183	0.06±0.08	0.11±0.15	0.13±0.11	0.0451	0.896
25	Butanoic acid hexyl ester	2639-63-6	12.92	2.11	1187.3	0.01±0.02	0.01±0.01	0.01±0.01	0.143	0.004
26	Octanoic acid ethyl ester	106-32-1	13.00	2.11	1195.9	47.4±90.81	0.39±0.61	3.64±6.44	2.6831	0.003
27	Methyl salicylate	119-36-8	13.08	2.82	1196.6	6.78±5.41	15.13±11.99	14.33±2.72	0.6708	0.902
28	Acetic acid octyl ester	112-14-1	13.33	2.09	1205.1	0.27±0.27	3.29±3.26	0.26±0.3	0.9412	0.013
29	3-Methyl butanoic acid hexyl ester	10032-13-0	13.75	2.05	1238.4	0±0.01	0±0.01	nd	0.0326	0.287
30	Benzeneacetic acid ethyl ester	101-97-3	13.92	2.83	1243.9	0.26±0.32	nd	nd	0.1222	0.134
31	2-Phenylethyl acetic acid ester	103-45-7	14.17	2.80	1258.1	0.02±0.02	2.96±2.77	0.28±0.18	1.0105	0
32	2-Hydroxy benzoic acid ethyl ester	118-61-6	14.50	2.69	1277.1	0.08±0.1	nd	0.01±0.01	0.1825	0.008
33	Hexanoic acid pentyl ester	540-07-8	14.67	2.11	1286.1	nd	0.03±0.02	0.05±0.04	0.053	0.241
34	3-Methyl-2-butenyl hexanoic acid ester	76649-22-4	14.67	2.20	1286.1	0.02±0.04	0.03±0.06	0.28±0.29	0.2508	0.006
35	Nonanoic acid ethyl ester	123-29-5	14.83	2.10	1295.6	0.5±0.39	nd	nd	0.0063	-
36	5-Methyl-2-(1-methylethyl)-cyclohexanol acetate	16409-45-3	14.83	2.23	1295.7	0.01±0.02	0.05±0.04	0.14±0.22	0.1587	0.088
37	Propanoic acid octyl ester	142-60-9	14.92	2.11	1300.3	nd	0.01±0.01	0±0.01	0.0445	0.034
38	Acetic acid nonyl ester	143-13-5	15.08	2.11	1309.8	0.01±0.02	0.05±0.06	0.03±0.03	0.0678	0.601
39	Decanoic acid methyl ester	110-42-9	15.33	2.11	1324.1	0.2±0.21	0.13±0.12	0.03±0.03	0.2354	0.118
40	3,7-Dimethyl-2,6-octadienoic acid methyl ester	2349-14-6	15.33	2.34	1324.4	0.05±0.05	0.07±0.09	0.21±0.16	0.2563	0.004
41	trans-8-p-Menthen-2-yl acetate	57287-13-5	15.42	2.15	1329.1	0.01±0.01	0.34±0.21	nd	0.2104	0.061
42	Carveol acetate	7111-29-7	15.50	2.41	1334	0.03±0.02	1.18±1.55	0.07±0.07	0.5059	0
43	Triacetin	102-76-1	15.50	2.83	1334.3	nd	12.11±21.32	11.63±21.6	1.5724	0.814
44	Citronellyl acetate	150-84-5	15.75	2.18	1348	0.03±0.04	9.86±17.27	0.39±0.29	1.3347	0.086

45	Neryl acetate*	141-12-8	15.92	2.33	1357.7	0.35±0.3	29.32±15.47	12.76±10.3	3.1067	0
46	(Z)-Hexanoic acid 3-hexenyl ester	31501-11-8	16.25	2.20	1376.6	0.08±0.06	0.19±0.25	0.28±0.21	0.086	0.363
47	Geranyl acetate*	105-87-3	16.25	2.34	1376.7	0.54±0.38	60.63±30.3	17.48±18.7	4.8095	0
48	Hexanoic acid hexyl ester	6378-65-0	16.33	2.12	1381.3	0.03±0.05	nd	0.03±0.02	0.1686	0.013
49	Decanoic acid ethyl ester*	110-38-3	16.50	2.11	1390.8	69.54±86.97	0.09±0.1	0.03±0.01	4.2634	0
50	Acetic acid decyl ester	112-17-4	16.75	2.09	1405.6	0.02±0.04	0.04±0.04	0.03±0.03	0.0434	0.547
51	p-Mentha-1,8-dien-7-yl acetate	15111-96-3	16.83	2.51	1411.3	0.13±0.11	1.03±1.03	0.12±0.14	0.4771	0
52	Coumarin*	91-64-5	17.50	3.96	1448	7.95±11.93	0.08±0.14	0.18±0.37	2.6553	0.043
53	2-Methyl-propanoic acid nonyl ester	10522-34-6	17.92	1.89	1473.8	0.02±0.03	nd	0.04±0.05	0.0647	0.399
54	Dihydroactinidiode	15356-74-8	18.92	3.44	1545.1	0.13±0.02	0.32±0.09	0.43±0.64	0.1991	0.002
55	3-Hexen-1-ol benzoate	72200-74-9	19.42	2.69	1573.1	0.05±0.04	0.06±0.06	0.02±0.02	0.1657	0.032
56	Dodecanoic acid ethyl ester	106-33-2	19.58	2.09	1578.8	0.33±0.28	0±0.01	nd	0.2659	0.087
57	2,2,4-Trimethyl-1,3-pentanediol diisobutyrate	6846-50-0	19.58	2.22	1589.2	0.01±0.01	0.04±0.03	0.03±0.03	0.0534	0.145
58	Octanoic acid phenylmethyl ester	10276-85-4	21.83	2.64	1700.8	1.49±2.34	0.03±0.07	nd	0.4137	0.796
59	Diisobutyl phthalate	84-69-5	23.33	2.87	1863.4	0.01±0.01	0±0.01	0.01±0.01	0.0257	0.485
60	E-11-Hexadecenoic acid ethyl ester	766512-32-7	24.67	2.20	1868	0.04±0.03	nd	nd	0.0306	0.117
	**Alkenes**									
61	1,3-trans-5-Octatriene	40087-61-4	6.67	1.96	879.41	0.01±0.01	0.18±0.05	0.3±0.46	0.1854	0.001
62	2-Methyl-5-(1-methylethyl)-bicyclo[3.1.0]hex-2-ene	2876-05-2	7.67	1.88	928.15	1.31±1.48	2.63±2.42	1.31±1.13	0.3428	0.673
63	α-Pinene*	80-56-8	7.83	1.93	936.17	12.42±7.51	15.81±13.49	9.43±8.92	0.6624	0.269
64	3-Ethyl-1,4-hexadiene	2080-89-9	8.00	2.05	944.29	0.05±0.02	0.31±0.28	0.08±0.06	0.2682	0
65	Camphene	79-92-5	8.25	1.98	956.22	1.16±1.57	1.12±0.57	0.55±0.34	0.4203	0.168
66	β-Pinene*	127-91-3	8.42	1.80	984.28	nd	0.03±0.07	0.02±0.05	0.0411	0.392
67	Sabinene	3387-41-5	8.67	2.00	976.25	2.4±2.11	27.23±35.12	5.47±13.43	1.6017	0.975

68	2,6-Dimethyl-2,6-octadiene	2792-39-4	8.83	1.90	984.14	nd	0.03±0.06	0.02±0.03	0.0874	0.402
69	β-Myrecene*	123-35-3	8.92	2.01	992.24	47.46±24.27	57.29±77.55	42.5±45.89	2.5033	0.512
70	1-Decene	872-05-9	9.00	1.77	992.07	0.01±0.02	nd	0.02±0.02	0.0412	0.499
71	3-Methyl-6-(1-methylethyl)-cyclohexene	5256-65-5	9.75	1.97	1028.2	0.03±0.01	0.06±0.07	0.04±0.03	0.0973	0.101
72	α-Terpinene*	99-86-5	9.75	2.07	1028.3	0.01±0.01	0.04±0.04	nd	0.18	0.016
73	Limonene*	138-86-3	9.83	2.33	1031	354.13±119.28	261.02±83.15	294.38±41.99	5.5397	0.025
74	(Z)-1,3,6-octatriene-3,7-dimethyl	3338-55-4	10.17	2.04	1048.3	5.05±4.76	49.03±25.84	4.71±0.89	4.8397	0
75	p-Mentha-1,5,8-triene	21195-59-5	10.75	2.31	1080.3	nd	0.18±0.05	0.12±0.22	0.1235	0.176
76	Terpinolene	586-62-9	11.00	2.16	1088.4	5.11±6.37	12.79±3.31	8.7±6.56	0.8139	0.303
77	3,3-Dimethyl-1-hexene	3404-77-1	11.25	1.78	1100.1	nd	0.18±0.2	0.45±0.88	0.3697	0.017
78	(E)-4,8-Dimethylnona-1,3,7-triene	19945-61-0	11.50	2.04	1113.3	0.23±0.13	0.08±0.11	0.11±0.08	0.1562	0.008
79	(E,E)-2,6-Dimethyl-1,3,5,7-octatetraene,	460-01-5	11.50	2.33	1117.9	0.41±0.15	3.27±1.21	0.54±0.16	1.1743	0
80	Decahydro-2-methyl naphthalene	2958-76-1	11.67	2.03	1122	0.01±0.02	nd	0.02±0.03	0.1428	0.027
81	2,6-Dimethyl-2,4,6-octatriene	673-84-7	12.00	2.15	1143.8	0.07±0.05	1.74±0.57	0.2±0.12	0.9164	0
82	1,3,8-p-Menthatriene	18368-95-1	12.00	2.37	1144	0.79±0.49	0.61±0.14	0.64±0.35	0.221	0.347
83	3,3,6,6-Tetramethyl-1,4-cyclohexadiene	2223-54-3	12.50	2.44	1165.8	0.02±0.02	0.01±0.01	0.14±0.13	0.2647	0.018
84	(3E,5Z)-1,3,5-Undecatriene	51447-08-6	12.67	2.05	1174.2	0.04±0.06	0.04±0.07	nd	0.1265	0.02
85	1,3,5,8-Undecatetraene	50277-31-1	12.67	2.14	1174.3	0.02±0.03	nd	nd	0.14	0.02
86	1-Dodecene	112-41-4	13.00	1.81	1191.4	0.09±0.05	0.29±0.28	0.18±0.13	0.2073	0.11
87	2,4-Dimethyl-2,6-octadiene	63843-03-8	14.50	2.38	1276.8	0.01±0.01	0.64±1.16	nd	0.7509	0.02
88	4-Ethenyl-4-methyl-3-(1-methylethenyl)-1-(1-methylethyl)-, (3R-trans)-cyclohexene	20307-84-0	15.67	2.08	1343.1	0.95±0.81	0.54±0.52	0.21±0.31	0.3447	0.212
89	3-Cyclohexen-1-yl-benzene	4994-16-5	15.67	2.71	1343.7	0.07±0.1	0.11±0.11	0.26±0.41	0.1218	0.355
90	3-Methylene-tridecane	19780-34-8	16.33	1.87	1381	0.01±0.01	0.01±0.02	0.03±0.02	0.0775	0.165

91	Copaene*	3856-25-5	16.42	2.12	1386	10.08±11.6	0.64±0.8	1.37±1.35	1.4689	0.002
92	(Z)-7-Tetradecene	41446-60-0	16.50	1.86	1390.5	0.1±0.04	0.13±0.1	0.16±0.04	0.0952	0.459
93	β-Elemene	515-13-9	16.58	2.17	1395.6	1.57±0.77	2.77±4.56	1.27±1.18	0.2707	0.134
94	(1S,5S)-2-Methyl-5-((R)-6-methylhept-5-en-2-yl)bicyclo[3.1.0]hex-2-ene	159407-35-9	16.75	2.06	1405.6	0.27±0.19	0.03±0.05	0.01±0.01	0.2179	0.01
95	5-Ethyl-1-nonene	19780-74-6	16.83	1.83	1410.6	0.05±0.06	nd	0.04±0.08	0.3511	0.036
96	Spiro[2.4]heptane, 1,5-dimethyl-6-methylene	62238-24-8	17.00	2.44	1421.8	0.01±0.02	nd	0.02±0.03	0.0078	0.67
97	trans-α-Bergamotene	13474-59-4	17.17	2.26	1437.2	8.41±9.81	6.29±3.55	9.63±7.83	0.8452	0.788
98	Caryophyllene	87-44-5	17.17	2.23	1432.1	10±8.04	6.83±12.22	7.74±9.44	1.8896	0.057
99	(E)-β-Famesene	18794-84-8	17.50	2.11	1449.7	9.4±13.7	2.59±3.97	0.78±0.69	1.4922	0.255
100	(1R,3aS,8aS)-7-Isopropyl-1,4-dimethyl-1,2,3,3a,6,8a-hexahydroazulene	36577-33-0	17.50	2.26	1453	1.61±1.83	2.01±3.53	0.25±0.19	0.6301	0.047
101	2-Methyl-3-methylene-2-(4-methyl-3-pentenyl)-, (1S-exo)-bicyclo[2.2.1]heptane	511-59-1	17.75	2.19	1457.9	nd	0.32±0.21	0.58±0.4	0.2308	0.38
102	Germacrene D	23986-74-5	17.83	2.30	1468.8	2.47±5.2	0.24±0.41	0.2±0.36	0.8256	0.004
103	Humulene	6753-98-6	17.75	2.30	1453.1	3.79±0.82	1±1.26	1.7±1.3	0.9286	0.001
104	1-Ethenyl-1-methyl-2,4-bis(1-methylethenyl)-cyclohexane	110823-68-2	17.83	1.92	1469.6	0.22±0.33	nd	0.04±0.08	0.2191	0.354
105	(Z,E)-3,7,11-Trimethyl-1,3,6,10-dodecatetraene	26560-14-5	18.00	2.18	1489.8	nd	0.01±0.01	nd	0.0652	0.005
106	α-Farnesene	502-61-4	18.25	2.17	1500	2.31±5.27	10.39±5.81	13.98±10.59	1.354	0.002
107	Ledene	21747-46-6	18.33	2.31	1506	9.69±4.27	1.52±2.21	1.54±0.78	1.7241	0
108	(Z)-1-Methyl-4-(6-methylhept-5-en-2-ylidene)cyclohex-1-ene	13062-00-5	18.50	2.26	1506.3	0.03±0.04	0.04±0.03	0.05±0.08	0.1422	0.862
109	Cadinene	483-76-1	18.67	2.34	1517.2	4.34±2.41	1.19±2.02	0.38±0.27	1.1614	0

110	(2S,4aR,8aR)-4a,8-Dimethyl-2-(prop-1-en-2-yl)-1,2,3,4,4a,5,6,8a-octahydronaphthalene	123123-37-5	18.75	2.39	1528.4	0.22±0.17	0.04±0.04	0.07±0.05	0.2044	0
111	α-Cadinene	24406-05-1	18.92	2.35	1534.9	0.52±0.32	0.2±0.31	0.11±0.12	0.3246	0.005
112	Selina-3,7(11)-diene	6813-21-4	19.08	2.37	1550.9	0.04±0.04	0.01±0.02	0±0.01	0.0659	0.076
113	(E,E)-1,5-Dimethyl-8-(1-methylethylidene)-1,5-cyclodecadiene	15423-57-1	19.33	2.47	1561.6	0.66±0.82	0.07±0.05	0.08±0.09	0.4045	0.016
114	8-Heptadecene	54290-12-9	20.92	1.89	1659.7	0.04±0.04	0.01±0.01	0.02±0.02	0.0876	0.041
115	Neophytadiene	504-96-1	23.00	1.93	1757.2	0.03±0.01	0.02±0.01	0.03±0.02	0.0611	0.007
	**Aldehydes**									
116	Pentanal	110-62-3	3.58	1.87	724.7	0.08±0.06	0.4±0.1	1.16±1.09	0.3454	0
117	2-Ethylacrolein	922-63-4	4.25	2.10	758.77	0.04±0.06	0.14±0.09	0.41±0.35	0.1413	0.001
118	(E)-2-Pentenal	1576-87-0	4.42	2.17	767.14	0.21±0.11	1.87±1.18	2.28±1.92	0.6326	0
119	3-Methyl-2-butenal	107-86-8	4.92	2.35	792.26	nd	0.07±0.04	0.08±0.06	0.1235	0.017
120	3-Hexenal	4440-65-7	5.08	2.15	800.45	0.02±0.03	0.26±0.17	0.39±0.39	0.2045	0.001
121	Hexanal*	66-25-1	5.08	2.12	800.4	0.35±0.12	2.16±1.28	6.5±5.64	0.785	0
122	2-Methyl-2-pentenal	623-36-9	5.42	2.23	821.32	nd	0.01±0.01	0.05±0.06	0.0017	0.059
123	2-Ethyl-trans-2-butenal	63883-69-2	5.67	2.23	829.67	nd	0.05±0.07	0.13±0.11	0.1987	0.062
124	2-Hexenal	505-57-7	6.08	2.38	854.74	2.8±2.06	19.62±13.03	29.42±25.17	1.8434	0
125	(Z)-4-Heptenal	6728-31-0	7.08	2.26	900.46	nd	0.05±0.05	0.14±0.19	0.0238	0.884
126	Heptanal*	111-71-7	7.08	2.17	900.39	0.15±0.21	0.19±0.13	0.58±0.65	0.0825	0.717
127	(E,E)-2,4-Hexadienal	142-83-6	7.33	2.62	912.75	0.57±0.12	5.3±4.7	2.5±1.75	1.164	0.003
128	(E)-2-Heptenal	18829-55-5	8.25	2.37	960.53	0.02±0.01	0.48±0.3	1.48±1.4	0.392	0
129	Benzaldehyde*	100-52-7	8.50	2.90	965.01	117.21±99.06	34.76±18.44	39.72±17.15	5.2562	0.112
130	(E,Z)-2,4-Heptadienal	4313-02-4	9.17	2.51	996.68	0.55±0.12	5.35±4.62	4.69±3.38	1.0656	0
131	Octanal*	124-13-0	9.25	2.21	1004.4	5.58±4.64	6.37±5.68	4.43±1.73	0.6174	0.512

132	(E,E)-2,4-Heptadienal*	4313-03-5	9.42	2.31	1012.7	2.29±0.61	20.28±18.15	18.34±22.04	2.0809	0.002
133	2,6-Dimethyl-5-heptenal	106-72-9	10.25	2.26	1052.4	0.1±0.15	nd	0.05±0.03	0.291	0.013
134	(E)-2-Octenal	2548-87-0	10.42	2.34	1060.5	0.05±0.02	0.38±0.33	1.38±1.61	0.3342	0
135	Benzeneacetaldehyde*	122-78-1	10.50	2.85	1064.9	1.4±1.13	3.79±1.17	35.37±37.18	2.7049	0
136	3-Methyl-benzaldehyde	620-23-5	11.00	2.92	1088.9	0.02±0.02	0.03±0.01	0.03±0.02	0.0563	0.305
137	Nonanal	124-19-6	11.25	2.21	1104.8	4.1±5.84	1.01±0.86	2.2±1.33	0.7039	0.21
138	(E,E)-2,4-Octadienal	30361-28-5	11.50	2.51	1109.4	0.13±0.05	0.68±0.38	1.02±1.45	0.3136	0
139	7-methyl-3-methylene-6-Octenal,	55050-40-3	12.00	2.39	1144	0.27±0.36	0.45±0.35	0.58±0.62	0.7826	0.003
140	(R)-3,7-Dimethyl-6-octenal	2385-77-5	12.17	2.07	1152.6	3.07±4.58	0.01±0.03	1.4±2.2	0.7632	0.029
141	(E,Z)-2,6-Nonadienal	557-48-2	12.25	2.46	1152.8	0.01±0.01	0.18±0.12	0.43±0.48	0.1973	0.001
142	(E)-2-Nonenal	18829-56-6	12.50	2.31	1161.4	0.03±0.03	0.09±0.08	0.5±0.65	0.247	0.001
143	3,5-Dimethyl-Benzaldehyde	5779-95-3	12.75	2.85	1179.2	0.05±0.03	0.22±0.05	0.32±0.36	0.1843	0
144	4-Decenal	21662-09-9	13.08	2.26	1196.1	0.21±0.32	nd	nd	0.3281	0.05
145	2,6,6-Trimethyl-1,3-Cyclohexadiene-1-carboxaldehyde	116-26-7	13.17	2.74	1205.7	0.05±0.03	0.16±0.09	0.9±1.2	0.3071	0.02
146	Decanal*	112-31-2	13.25	2.25	1205.2	27.34±33.72	0.34±0.2	2.83±3.59	2.3507	0.089
147	(E,E)-2,4-Nonadienal	5910-87-2	13.50	2.48	1219.7	0.1±0.03	0.37±0.32	0.51±0.47	0.2289	0.003
148	β-Cyclocitral	432-25-7	13.58	2.62	1224.6	0.04±0.06	0.04±0.07	0.5±0.77	0.2441	0.262
149	Neral	106-26-3	13.83	2.52	1243.5	5.11±6.55	3.42±3.17	77.85±76.55	6.6702	0.051
150	4-(1-Methylethyl)-benzaldehyde	122-03-2	14.00	2.77	1248.6	0.57±0.75	0.19±0.25	0.03±0.01	0.4054	0
151	β-Homcyclocitral	472-66-2	14.17	2.40	1262.6	0.01±0	nd	0.08±0.09	0.0838	0
152	(E)-3,7-Dimethyl-2,6-octadienal	141-27-5	14.33	2.55	1272.1	7.11±9.1	9.75±6.78	17.1±14.56	1.3008	0.182
153	Benzeneacetaldehyde, α-ethylidene-	4411-89-6	14.50	3.16	1277.5	0.14±0.19	0.1±0.05	0.61±0.6	0.271	0.021
154	3-Phenyl-2-propenal	104-55-2	14.67	3.19	1287.1	5.51±12.24	0.49±0.32	5.69±11.15	1.2036	0.288
155	Undecanal	112-44-7	15.08	2.15	1309.9	0.18±0.22	0.04±0.04	0.12±0.1	0.133	0.784
156	(E,E)-2,4-Decadienal	25152-84-5	15.33	2.43	1324.4	0.44±0.4	0.27±0.23	0.75±1.02	0.1307	0.675

157	Dodecanal	112-54-9	16.75	2.17	1405.7	5.74±8.48	0.05±0.03	0.18±0.21	1.0764	0.423
158	2,6-Dimethyl-10-methylene-2,6,11-dodecatrienal	60066-88-8	21.17	2.51	1689.5	0.07±0.06	0.01±0.01	0.01±0.01	0.1163	0.001
159	(E,E,E)-2,6,10-Trimethyl-2,6,9,11-dodecatetraenal	17909-77-2	22.00	2.76	1751	nd	0.01±0.02	0.01±0.01	0.099	0.347
160	2-Methyl-hexadecanal	55019-46-0	23.00	2.11	1840.1	0.23±0.05	0.23±0.16	0.2±0.19	0.2338	0
	**Ketones**									
161	(E)-3-Penten-2-one	3102-33-8	4.17	2.15	754.63	0.01±0.01	0.17±0.05	0.46±0.4	0.2298	0.001
162	2-Hexanone	591-78-6	4.92	2.07	792.05	nd	0.04±0.04	0.06±0.11	0.0154	0.405
163	4-Methyl-3-penten-2-one	141-79-7	5.08	2.22	800.48	nd	0.05±0.05	0.53±0.5	0.1797	0.254
164	2-Heptanone	110-43-0	6.83	2.17	887.92	0.15±0.06	0.51±0.18	1.47±1.52	0.3076	0
165	Cyclohexanone	108-94-1	7.00	2.74	896.72	0.03±0.04	nd	0.02±0.03	0.063	0.148
166	(E)-3-Hepten-2-one	5609-09-6	7.83	2.35	936.52	nd	nd	0.1±0.11	0.1927	0.008
167	6-Methyl-2-heptanone	928-68-7	8.17	2.17	952.38	nd	nd	0.1±0.18	0.199	0.037
168	1-Octen-3-one	4312-99-6	8.67	2.28	976.45	nd	0.06±0.05	0.42±0.46	0.258	0.306
169	3-Octanone	106-68-3	8.83	2.20	984.41	nd	0.07±0.12	0.09±0.12	0.1142	0.102
170	2-Octanone	111-13-7	8.92	2.24	988.43	0.01±0.02	0.04±0.03	0.2±0.3	0.1035	0.417
171	6-Methyl-5-hepten-2-one	110-93-0	8.83	2.35	984.52	0.35±0.11	1.98±1.19	5.76±5.5	0.8	0
172	Benzocyclobuten-1(2H)-one	3469-06-5	9.17	2.71	1000.8	0.06±0.09	0.05±0.01	0.07±0.07	0.1304	0.11
173	3-Octen-2-one	1669-44-9	10.00	2.35	1036.5	0.06±0.05	0.2±0.18	0.79±0.92	0.2951	0.049
174	Acetophenone	98-86-2	10.58	3.01	1069	0.28±0.14	0.49±0.24	0.55±0.56	0.2953	0.009
175	3,5-Octadien-2-one, isomer1	30086-02-3	10.75	2.52	1068.7	0.36±0.21	2.94±2.31	3±3.88	0.7791	0
176	3-Nonanone	925-78-0	10.92	2.17	1084.4	nd	nd	0.13±0.24	0.1816	0.076
177	2-Nonanone	821-55-6	11.00	2.20	1088.4	0.02±0.03	nd	0.22±0.27	0.1219	0.02
178	Fenchone	1195-79-5	11.08	2.47	1092.6	nd	0.1±0.08	0.25±0.29	0.3332	0.001
179	3,5-Octadien-2-one, isomer2	30086-02-3	11.08	2.55	1092.7	0.3±0.19	1.18±0.38	2.46±3.82	0.3387	0.001

180	6-Methyl-3,5-heptadiene-2-one	1604-28-0	11.33	2.61	1105.1	0.12±0.06	0.77±0.31	1±1.38	0.4495	0
181	3-Nonen-2-one	14309-57-0	11.92	2.34	1135.3	0.06±0.1	0.11±0.12	0.54±0.68	0.1823	0.413
182	4-Acetyl-1-methylcyclohexene	5259-65-4	11.92	2.60	1135.5	0.06±0.05	0.18±0.19	0.14±0.12	0.1311	0.465
183	(R,S)-5-Ethyl-6-methyl-3E-hepten-2-one	57283-79-1	12.00	2.26	1139.6	0.03±0.04	nd	0.47±0.85	0.4618	0.094
184	(1S)-1,7,7-Trimethyl-bicyclo[2.2.1]heptan-2-one	464-48-2	12.25	2.63	1157.3	0.03±0.02	0.25±0.16	0.04±0.05	0.3009	0.004
185	5-Methyl-2-(1-methylethyl)-cyclohexanone	10458-14-7	12.42	2.39	1161.4	0.02±0.01	0.32±0.43	0.85±1.54	0.5966	0.001
186	Pinocarvone	30460-92-5	12.58	2.65	1170.4	0.01±0.02	0.12±0.11	0.03±0.04	0.1888	0.007
187	1-(3-Methylphenyl)-ethanone	585-74-0	13.00	2.92	1192.3	0.22±0.19	0.74±0.4	0.28±0.08	0.5464	0
188	trans-2-Methyl-5-(1-methylethenyl)-cyclohexanone	5948-04-9	13.17	2.59	1200.8	0.23±0.19	0.08±0.08	0.09±0.09	0.1941	0.058
189	α,4-Dimethyl-3-cyclohexene-1-acetaldehyde	29548-14-9	13.58	2.57	1224.6	0.62±0.46	0.19±0.12	0.58±0.33	0.3324	0
190	(-)-Carvone	99-49-0	14.00	2.76	1248.6	8.27±11.19	1.5±1.34	2.43±1.31	1.1249	0.975
191	Piperitone	89-81-6	14.25	2.72	1262.8	0.07±0.06	0.09±0.11	0.3±0.1	0.2437	0.007
192	3-Methyl-6-(1-methylethenyl)-2-cyclohexen-1-one	529-01-1	14.50	2.95	1277.3	0.19±0.15	0.12±0.06	0.21±0.13	0.2459	0.021
193	3-Undecanone	2216-87-7	14.67	2.17	1286.1	2.21±3.35	0.25±0.27	0.45±0.79	1.1405	0.191
194	Paroxypropione	70-70-2	14.67	2.93	1291.6	0.01±0.02	0.04±0.07	0.02±0.05	0.1289	0.082
195	4-Isopropyl-7-methyl-3,8-dioxatricyclo[5.1.0.02,4]octane	1619-26-7	15.17	2.83	1315.3	nd	0.03±0.03	nd	0.0566	0.117
196	Piperitenone	491-09-8	15.75	3.02	1348.8	0.01±0	0.01±0.02	0.01±0.01	0.0817	0.021
197	Damascenone	23696-85-7	16.42	2.61	1386.5	0.14±0.15	0.04±0.02	0.87±1.21	0.3268	0
198	Jasmone	488-10-8	16.58	2.90	1396.3	nd	0±0.01	0.03±0.03	0.0893	0.134
199	6,10-Dimethyl-2-undecanone	1604-34-8	16.67	2.13	1400.3	0.03±0.02	0.03±0.03	0.1±0.17	0.0183	0.87

200	α-Ionone*	127-41-3	17.08	2.49	1427.1	0.11±0.06	0.16±0.09	0.41±0.64	0.0515	0.035
201	Geranyl acetone	3796-70-1	17.42	2.37	1443.1	0.94±0.18	1.52±0.85	3.51±5.34	0.2869	0.059
202	4-(2,6,6-Trimethylcyclohexa-1,3-dienyl)but-3-en-2-one	1203-08-3	17.92	2.69	1484.5	0.03±0	0.05±0.06	0.27±0.36	0.1104	0.161
203	trans-β-Ionone*	79-77-6	18.00	2.58	1479.9	0.26±0.1	0.42±0.17	0.99±1.34	0.0887	0.133
204	3-Tridecanone	1534-26-5	18.08	2.14	1485	nd	0.25±0.28	0.23±0.39	0.229	0.037
205	Benzophenone	119-61-9	20.33	3.49	1600.9	0.16±0.24	nd	0.01±0.02	0.297	0.05
	**Alcohols**									
206	1-Pentanol	71-41-0	4.58	1.96	775.3	0.05±0.02	0.17±0.05	0.41±0.34	0.1792	0
207	(Z)-2-Penten-1-ol	1576-95-0	4.58	2.06	775.36	0.12±0.1	0.55±0.2	1.6±1.62	0.369	0.001
208	3-Hexen-1-ol	544-12-7	6.17	2.20	854.63	0.01±0.01	0.14±0.09	0.16±0.21	0.177	0.001
209	(E)-2-Hexen-1-ol	928-95-0	6.42	2.15	867.08	0.06±0.11	nd	0.18±0.29	0.2049	0.018
210	1-Hexanol	111-27-3	6.42	2.10	871.18	0.6±0.89	0.07±0.07	2.59±3.64	0.6794	0.02
211	1-Heptanol	111-70-6	8.50	2.13	968.35	0.03±0.04	nd	0.01±0.01	0.13	0.042
212	1-Octen-3-ol	3391-86-4	8.75	2.10	980.32	0.01±0.01	0.13±0.15	0.53±0.69	0.4894	0.001
213	Benzyl alcohol	100-51-6	10.08	2.87	1044.9	4.45±6.59	1.05±1.36	1.34±1.34	1.1217	0.206
214	(1R,2R,5S- 2-Methyl-5-(1-methylethyl)-bicyclo[3.1.0]hexan-2-ol	17699-16-0	10.58	1.92	1072.4	0.06±0.05	0.14±0.08	0.18±0.21	0.2133	0.119
215	(E)-2-Octen-1-ol	18409-17-1	10.58	2.23	1068.4	nd	0.06±0.07	0.12±0.15	0.1057	0.041
216	trans-Linalool oxide	34995-77-2	10.67	2.20	1072.4	0.06±0.09	0.71±0.52	0.39±0.43	0.6209	0.001
217	1-Octanol	111-87-5	10.67	2.12	1072.3	0.15±0.16	0.45±0.51	0.32±0.26	0.1984	0.765
218	Linalool*	78-70-6	10.83	2.17	1084.4	24.08±8.83	42.02±25.89	15.89±7.49	4.1578	0
219	cis-Linalool oxide	5989-33-3	11.00	2.24	1088.4	0.17±0.07	0.88±0.36	0.65±0.96	0.3495	0.002
220	3,7-Dimethyl-1,5,7-octatrien-3-ol	29957-43-5	11.33	2.26	1100.5	0±0.01	0.53±0.58	0.6±0.86	0.2306	0.017
221	trans-1-Methyl-4-(1-methylethenyl)-2-cyclohexen-1-ol	7212-40-0	11.67	2.19	1126.6	nd	0.01±0.03	0.02±0.03	0.0959	0.076

222	Phenylethyl Alcohol	60-12-8	11.67	2.88	1122.8	0.09±0.06	0.92±0.75	3.68±4.83	0.5802	0
223	1,2-Dihydrolinalool	18479-51-1	11.92	2.12	1135.1	0.14±0.13	0.96±1.72	nd	0.5415	0.085
224	1-Methyl-4-(1-methylethenyl)-cyclohexanol	138-87-4	12.25	2.35	1152.7	nd	nd	0.01±0.02	0.0465	0.18
225	p-Mentha-1,5-dien-8-ol	1686-20-0	12.67	2.51	1174.6	0.02±0.03	0.04±0.05	0.15±0.1	0.1318	0.362
226	Levomenthol	2216-51-5	12.75	2.37	1183.1	0.05±0.07	0.05±0.09	0.66±0.49	0.2132	0.244
227	(3R,6S)-2,2,6-Trimethyl-6-vinyltetrahydro-2H-pyran-3-ol	39028-58-5	12.83	2.39	1178.8	0±0.01	nd	0.2±0.32	0.1356	0.02
228	Terpinen-4-ol*	562-74-3	12.92	2.35	1187.5	1.31±0.7	1.75±1.08	11.42±12.7	2.0878	0.037
229	2,6-Dimethyl-3,7-octadiene-2,6-diol	13741-21-4	13.00	2.40	1191.8	0.01±0.02	0.54±0.95	nd	0.2052	0.004
230	trans-p-Mentha-1(7),8-dien-2-ol	21391-84-4	13.00	2.48	1191.9	nd	0.01±0.01	0.01±0.03	0.1037	0.044
231	α,α,4-Trimethyl-benzenemethanol	1197-01-9	13.00	2.64	1192.1	0.11±0.09	0.77±0.51	0.5±0.29	0.4172	0.011
232	Terpineol	98-55-5	13.17	2.41	1200.6	5.06±3.57	31.18±19.88	17.36±14.65	2.0138	0
233	trans-Isopiperitenol	74410-00-7	13.33	2.47	1205.5	0.63±0.9	0.12±0.06	0.21±0.15	0.4265	0.562
234	2-Methyl-5-(1-methylethenyl)-2-cyclohexen-1-ol	1197-06-4	13.50	2.45	1224.5	0.34±0.44	0.15±0.1	0.19±0.13	0.1607	0.221
235	Nerol*	106-25-2	13.58	2.29	1229.1	0.1±0.09	3.16±1.79	1.34±1.55	1.1879	0
236	Citronellol	106-22-9	13.75	2.20	1229	nd	0.34±0.62	0.04±0.07	0.3399	0.247
237	Carveol	99-48-9	13.83	2.55	1238.8	0.32±0.4	0.28±0.16	5.06±4.98	1.6102	0.317
238	2,6-Dimethyl-1,7-octadiene-3,6-diol	51276-33-6	14.67	2.59	1281.7	0±0	1.02±2.22	0.29±0.34	0.1634	0.289
239	Perillal	2111-75-3	14.67	2.78	1286.7	9.93±16.12	0.14±0.14	0.36±0.2	1.4911	0
240	p-Mentha-1(7),8(10)-dien-9-ol	29548-13-8	15.00	2.58	1300.8	0.02±0.02	0.01±0.01	nd	0.0394	0.229
241	Nerolidol	142-50-7	19.17	2.25	1556.2	0.13±0.05	0.13±0.1	0.13±0.06	0.134	0.074
242	Cedreanol	19435-97-3	20.58	2.57	1659	0.37±0.44	0.1±0.17	0.01±0.02	0.3028	0.014
	**Aromatic hydrocarbons**									
243	Toluene	108-88-3	4.58	2.00	774.89	0.2±0.12	1.27±0.56	3.24±2.37	0.7626	0

244	Ethylbenzene	100-41-4	6.33	2.16	862.92	0.02±0.02	0.33±0.47	0.83±1.07	0.2486	0.021
245	1,3-Dimethylbenzene	108-38-3	6.50	2.15	871.24	0.2±0.31	0.73±0.58	1.45±1.37	0.2902	0.003
246	o-Xylene	95-47-6	6.83	2.07	887.92	0.17±0.33	0.19±0.26	0.7±0.91	0.1085	0.177
247	Styrene	100-42-5	7.00	2.33	896.38	0.24±0.37	0.18±0.08	0.31±0.23	0.4093	0.015
248	β-Methylstyrene	98-83-9	8.83	2.43	984.55	nd	0.03±0.04	0.06±0.1	0.1043	0.102
249	1,2,3-Trimethylbenzene	526-73-8	9.67	2.39	1024.5	0.03±0.02	0.15±0.1	1.41±2.29	0.3289	0.005
250	p-Cymene	99-87-6	9.75	2.20	1028.4	28.18±23.45	69.33±84.62	57.96±28.56	2.2795	0.19
251	o-Cymene*	527-84-4	9.75	2.30	1028.4	19.39±29.2	26.56±32.24	nd	2.2645	0.013
252	1-Methyl-4-(1-methylethenyl)-benzene*	1195-32-0	11.08	2.41	1092.5	4.59±4.82	6.92±3.97	6.12±2.28	0.2886	0.348
253	2,3-Dihydro-4-methyl-1H-indene	824-22-6	12.08	2.52	1144.2	nd	0±0.01	0.02±0.03	0.0148	0.513
254	1-Methyl-3-(1-methylethyl)-benzene	535-77-3	12.25	2.48	1157.2	0.03±0.01	0.07±0.03	0.56±0.88	0.2184	0.016
255	(3-Methylbutyl)-benzene	2049-94-7	12.33	2.23	1161.3	0.01±0.01	nd	0.04±0.06	0.092	0.042
256	1,3-Bis(1,1-dimethylethyl)-benzene	1014-60-4	14.08	2.14	1252.7	nd	nd	0.13±0.23	0.0053	
257	1-Methyl-naphthalene	90-12-0	15.08	2.95	1310.6	0.09±0.03	0.23±0.14	0.12±0.05	0.2219	0.003
258	Biphenyl	92-52-4	16.50	3.04	1391.7	0.05±0.02	0.05±0.01	0.03±0.01	0.1333	0
259	Calamenene	483-77-2	18.75	2.48	1534	5.25±6.93	0.74±1.21	0.27±0.1	1.0595	0.002
260	α-Calacorene	21391-99-1	19.00	2.61	1544.4	0.49±0.5	0.17±0.25	0.08±0.04	0.3248	0.003
261	1,2,3,4-Tetrahydronaphthalene4-isopropyl-6-methyl-1-methylene	50277-34-4	19.33	2.64	1572.9	0.08±0.07	0.02±0.04	0.01±0.01	0.1889	0.008
262	1,6-Dimethyl-4-(1-methylethyl)-naphthalene	483-78-3	21.00	2.85	1682.8	0.13±0.16	0.04±0.06	0.02±0.02	0.1457	0.009
	**Oxyheterocyclic compounds**									
263	2-Ethylfuran*	3208-16-0	3.58	1.77	724.39	0.13±0.1	3.27±2.18	3.24±2.77	0.829	0
264	2,5-Dimethyl-furan	625-86-5	3.67	1.78	729.36	nd	nd	0.04±0.05	0.052	0.265
265	2-Vinylfuran	1487-18-9	3.92	1.90	741.96	nd	0.01±0.01	0.04±0.03	0.0416	0.047
266	2-Propyl-furan	4229-91-8	4.92	1.92	791.93	nd	nd	0.04±0.07	0.1092	0.079

267	3-Furaldehyde	498-60-2	5.42	2.64	817.51	nd	0.09±0.09	0.13±0.14	0.0231	0.409
268	Furfural*	98-01-1	5.75	2.85	834.3	0.89±0.52	3.06±1.58	42.28±50.4	2.695	0
269	2-n-Butyl furan	4466-24-4	6.92	2.02	891.95	0.01±0.01	0.08±0.03	0.31±0.49	0.1481	0.003
270	1-(2-Furanyl)-ethanone	1192-62-7	7.33	2.84	912.93	0.08±0.06	0.39±0.19	3.8±4	0.7411	0.005
271	2,7-Dimethyl-oxepine	1487-99-6	7.75	2.23	932.43	nd	0.03±0.02	0.09±0.11	0.0196	1
272	2-Pentyl-furan	3777-69-3	9.00	2.12	992.33	0.58±0.22	2.59±0.91	9.98±14.78	0.8039	0
273	trans-2-(2-Pentenyl)furan	70424-14-5	9.17	2.20	1000.4	0.05±0.04	0.41±0.26	0.97±1.4	0.2436	0.001
274	1-(2-Furanyl)-1-propanone	3194-15-8	9.33	2.81	1008.9	nd	0.02±0.02	0.13±0.16	0.0497	0.289
275	3,4-Dimethyl-2,5-furandione	766-39-2	9.83	3.35	1033.3	nd	0±0.01	0.15±0.2	0.1338	0.201
276	2-Acetyl-5-methylfuran	1193-79-9	9.92	2.93	1037	nd	0.01±0.01	0.04±0.07	0.1	0.02
277	5-Ethyl-2-furaldehyde	23074-10-4	10.33	2.91	1057	nd	0.08±0.06	0.13±0.25	0.1804	0.106
278	3-Methyl-2-(2-methyl-2-butenyl)-furan	15186-51-3	11.08	2.25	1092.4	0.02±0.04	0.1±0.19	0.17±0.17	0.107	0.839
279	4,7-Dimethyl-benzofuran	28715-26-6	13.58	2.66	1224.7	0.17±0.15	0.17±0.13	0.13±0.08	0.1361	0.31
280	3-Phenyl-furan	13679-41-9	13.67	2.88	1229.7	0.27±0.27	0.32±0.09	0.76±0.17	0.353	0.01
281	2-Heptyl-4-methyl-1,3-dioxolane	74094-61-4	18.17	2.18	1489.8	0.01±0.01	0.02±0.02	0.01±0.02	0.0969	0.025
282	Dibenzofuran	132-64-9	18.75	3.25	1534.1	0.03±0.05	0.01±0.01	nd	0.0779	0.012
	**Nitrogen-containing compounds**									
283	Pyrazine	290-37-9	4.17	2.32	754.78	nd	0.02±0.03	0.31±0.4	0.3286	0.004
284	1-Ethyl-1H-pyrrole	617-92-5	5.33	2.33	813.08	nd	0.05±0.05	0.44±0.6	0.2256	0.01
285	Methyl-pyrazine	109-08-0	5.67	2.52	829.88	0.03±0.05	0.07±0.1	0.73±1.14	0.3284	0.153
286	2,5-Dimethyl-pyrazine	123-32-0	7.42	2.53	916.69	nd	0.02±0.04	0.3±0.62	0.4061	0.121
287	Benzonitrile	100-47-0	8.92	3.07	989.09	nd	0.05±0.03	0.35±0.63	0.3128	1
288	2-Ethyl-5-methyl-pyrazine	13360-64-0	9.25	2.57	1004.7	nd	nd	0.17±0.3	0.2752	0.05
289	1H-Pyrrole-2-carboxaldehyde	1003-29-8	9.50	3.12	1017.1	nd	0.02±0.03	0.13±0.23	0.1702	0.332
290	1-Nitro-hexane	646-14-0	10.08	2.54	1044.6	nd	0.05±0.1	0.06±0.12	0.0244	0.682
291	1-(1H-pyrrol-2-yl)-ethanone	1072-83-9	10.50	3.14	1069.1	0.02±0.04	0.15±0.16	1.11±1.81	0.3734	0.039

292	Pyrazine, 3-ethyl-2,5-dimethyl-	13360-65-1	10.75	2.56	1076.7	nd	nd	0.05±0.09	0	-
293	N-ethylsuccinimide	2314-78-5	11.92	3.59	1136.4	0.06±0.04	0.1±0.07	0.25±0.37	0.0459	0.389
294	Benzyl nitrile	140-29-4	12.08	3.36	1144.9	0.03±0.04	0.15±0.2	0.12±0.07	0.1893	0.237
295	1-(2-Furanylmethyl)-1H-pyrrole	1438-94-4	12.75	3.00	1179.3	nd	nd	0.21±0.37	0.3047	0.05
296	(Isocyanomethyl)-benzene	10340-91-7	12.83	2.69	1183.4	0.01±0.02	0.01±0.02	0.02±0.03	0.0538	0.994
297	3-Ethyl-4-methyl-1H-pyrrole-2,5-dione	20189-42-8	13.75	3.05	1239.3	0.01±0.01	0.04±0.03	0.07±0.12	0.1261	0.012
298	Indole	120-72-9	15.00	3.55	1306.4	0.01±0.02	0.4±0.49	0.04±0.05	0.218	0.04
299	Methyl anthranilate	134-20-3	15.83	3.28	1353.8	0.06±0.08	0.17±0.18	0.02±0.02	0.186	0.027
300	Benzoic acid, 2-(methylamino)-, methyl ester	85-91-6	16.92	3.10	1417.2	0.39±0.39	0.45±0.7	0.05±0.05	0.2867	0.063
301	Caffeine	58-08-2	23.25	4.45	1840.3	0.44±0.35	0.33±0.16	0.63±0.44	0.1473	0.174
	**Alkanes**									
302	Pentane	109-66-0	2.25	3.97	660.34	0.01±0.01	0.07±0.05	0.05±0.08	0.1137	0.021
303	Octane	111-65-9	5.08	1.62	800	0.02±0.02	0.08±0.03	0.16±0.14	0.0778	0.048
304	2,4-Dimethyl-heptane	2213-23-2	5.50	1.62	820.83	0.01±0.01	0.07±0.13	0.09±0.09	0.2488	0.058
305	4-Methyl-octane	2216-34-4	6.33	1.65	862.51	nd	0.03±0.05	0.07±0.06	0.1102	0.152
306	Propyl-cyclopropane	2415-72-7	7.67	2.06	928.3	0.01±0.01	nd	0.06±0.06	0.1309	0.02
307	2-Methyl-nonane	871-83-0	8.42	1.69	968	nd	0.01±0.02	0.04±0.05	0.0014	0.739
308	trans-p-Menthane	1678-82-6	8.83	1.90	984.12	nd	0.02±0.03	0±0.01	0.1484	0.02
309	2,2,4,6,6-Pentamethyl-heptane	13475-82-6	9.00	1.69	991.99	0.03±0.02	0.05±0.07	0.13±0.05	0.0922	0.32
310	Pentyl-cyclohexane	4292-92-6	12.00	1.90	1139.3	0.01±0.01	nd	0±0.01	0.1487	0.027
311	2,6-Dimethyl-undecane	17301-23-4	13.33	1.74	1209.5	0.1±0.1	0.13±0.04	0.16±0.07	0.1333	0.084
312	4,8-Dimethyl-undecane	17301-33-6	13.50	1.74	1219	0.01±0.01	0.04±0.03	0.04±0.04	0.0948	0.002
313	1,1-Diethoxy-octane	54889-48-4	14.58	1.94	1281.1	1.79±2.73	nd	0.01±0.02	1.4264	0.02
314	5-Methyl-tridecane	25117-31-1	15.83	1.77	1352.4	0.12±0.04	0.12±0.04	0.14±0.06	0.1275	0.034
315	1-Hexyl-3-methyl-cyclopentane	61142-68-5	16.67	1.89	1400.1	nd	nd	0.01±0.01	0.024	0.6

316	2,6,10-Trimethyltridecane	3891-99-4	17.58	1.78	1453.1	0.1±0.03	0.2±0.09	0.27±0.13	0.141	0.003
317	2,7,10-Trimethyl-dodecane	74645-98-0	18.25	1.80	1495.1	0.2±0.05	0.31±0.15	0.54±0.16	0.1862	0.161
318	2,9-Dimethyl-decane	1002-17-1	18.92	1.80	1546.3	0.09±0.03	0.08±0.03	0.17±0.07	0.0212	0.561
319	Decylcyclopentane	1795-21-7	20.58	1.96	1643.1	0.01±0.01	nd	0.02±0.02	0.0156	0.723
	**Ethers**									
320	2-Ethenyltetrahydro-2,6,6-trimethyl-2H-pyran	7392-19-0	8.58	1.97	972.22	0.02±0.02	0.07±0.07	0.08±0.09	0.0679	0.225
321	(2R,5R)-2-Methyl-5-(prop-1-en-2-yl)-2-vinyltetrahydrofuran	54750-70-8	9.00	2.12	992.35	nd	0.21±0.19	nd	0.0169	
322	1-Methyl-4-(1-methylethyl)-7-oxabicyclo[2.2.1]heptane	470-67-7	9.42	2.25	1020.3	0.21±0.31	4.05±2.93	2.03±2.12	0.5951	0.112
323	1-Methoxy-octane	929-56-6	9.75	2.11	1028.2	nd	nd	0±0.01	0.0186	0.05
324	1,2-Dimethoxy-benzene	91-16-7	12.08	3.02	1144.6	nd	0.01±0.01	nd	0.0005	-
325	1-Ethenyl-4-methoxy-benzene	637-69-4	12.50	2.83	1166	nd	0.01±0.02	0.04±0.08	0.1271	0.036
326	Estragole	140-67-0	13.17	2.63	1200.8	0.11±0.15	2.73±4.42	0.54±0.76	0.6225	0.029
327	2-Methoxy-4-methyl-1-(1-methylethyl)-benzene	1076-56-8	13.67	2.43	1234	0.08±0.11	nd	nd	0.0061	-
328	Anethole	104-46-1	14.83	2.74	1296.2	7.76±11.68	4.39±4.16	4.62±5.91	0.7902	0.57
329	Safrole	94-59-7	14.92	2.79	1296.2	nd	0.18±0.19	0.11±0.13	0.1919	0.12
330	Theaspirane A	66537-39-1	15.08	2.28	1310	0.04±0.02	0.06±0.03	0.21±0.28	0.1011	0.505
331	trans-3,5,6,8a-Tetrahydro-2,5,5,8a-tetramethyl-2H-1-benzopyran	41678-29-9	15.25	2.43	1319.7	0.01±0	0.01±0.02	0.17±0.29	0.181	0.735
332	Theaspirane B*	36431-72-8	15.33	2.33	1324.3	0.03±0.02	0.05±0.03	0.22±0.3	0.0924	0.313
333	Precocene I	17598-02-6	17.75	2.93	1469	0.01±0.01	0.04±0.03	0.13±0.21	0.0686	0.029
334	Caryophyllene oxide	1139-30-6	19.75	2.60	1589.4	1.12±0.63	0.7±0.89	0.3±0.23	0.5143	0.026
	**Phenols**									

335	Phenol	108-95-2	8.83	2.65	984.75	nd	0.08±0.06	0.1±0.1	0.0886	0.184
336	p-Cresol	106-44-5	10.75	2.70	1076.8	nd	0.18±0.22	0.05±0.05	0.192	0
337	Thymol	89-83-8	14.67	2.61	1291.3	0.01±0.02	0.41±0.54	0.08±0.11	0.197	0.079
338	2-Methyl-5-(1-methylethyl)-phenol	499-75-2	15.00	2.65	1305.6	0.74±1.11	1.02±1.29	0.27±0.46	0.511	0.189
339	2-Methoxy-3-(2-propenyl)-phenol	1941-12-4	15.92	2.88	1358.2	0.02±0.03	0.77±1.14	0.01±0.02	0.6986	0.005
340	Butylated Hydroxytoluene	128-37-0	18.33	2.43	1500.6	nd	0.05±0.09	0.37±0.63	0.1915	0.522
341	2,4-Di-tert-butylphenol	96-76-4	18.33	2.47	1506.3	0.02±0.01	0.03±0.02	0.15±0.18	0.1417	0.003
	**Acids**									
342	Hexanoic acid	142-62-1	8.67	2.11	976.35	0.09±0.15	0.4±0.29	4.11±4.23	0.7674	0.405
343	2-Hexenoic acid	1191-04-4	9.50	2.26	1012.4	0±0	0.02±0.04	0.4±0.55	0.313	0.011
344	Nonanoic acid	112-05-0	14.33	2.15	1267	0.07±0.04	0.07±0.08	0.37±0.45	0.1642	0.906
345	(E)-2,6-Octadienoic acid, 3,7-dimethyl-	4698-08-2	15.75	2.43	1348.2	0.15±0.23	nd	0.33±0.3	0.1588	0.061
346	n-Decanoic acid	334-48-5	16.00	2.18	1362.3	0.87±0.99	0.22±0.25	0±0.01	0.4279	0.005
	**Sulfur-compounds**									
347	N-Morpholinomethyl-isopropyl-sulfide	77422-34-5	20.92	2.22	1676.5	0.04±0.02	0.03±0.01	0.01±0.02	0.0339	0.135
	**Alkynes**									
348	1-Propynyl-benzene	673-32-5	10.17	2.75	1048.8	nd	0.03±0.03	0.01±0.02	0.1123	0.002

Note: [1] RT: retention time; *: the compounds were identified by authentic standards; nd: not detected; [2] The data was showed as the mean value ± standard deviation; [3] The value was calculated in UV-scaling form of the PLS-DA model; [4] The P-Value was calculated by Tukey s-b(K) test.

Table S3. The detailed performance of GC-O analysis of Citrus Group samples.

			Assessor 1		Assessor 2		Assessor 3		Assessor 4		Assessor 5		Assessor 6		Assessor 7	
**NO.**	**Compounds**	**Class**	**Aroma characteristics**	**AI[1]**	**Aroma characteristics**	**AI**	**Aroma characteristics**	**AI**	**Aroma characteristics**	**AI**	**Aroma characteristics**	**AI**	**Aroma characteristics**	**AI**	**Aroma characteristics**	**AI**
1	Ethyl butyrate	B	Fruity	2	Fruity	3	Fresh, Fruity	2	Fruity	3	Sweet	3	Floral	2		
2	2-Hexenal	B	Fruity, Fresh	2	Fruity	2	Fresh	2	Fatty cheesy	3	Sweet, Fresh	2	Sweet, Fruity	3	Fresh	3
3	Heptanal	A	Pungent, Fatty	3	Fatty, Fresh	1			Fresh, Green	4	Fresh, Green	2	Floral, Fatty	3		
4	β-Myrecene	A	Plastic	3	Green, Mushroom-like	2	Fresh peppery, Green	3	Fresh, Metallic	4	Green, Fatty, Metallic	3	Fresh, Green, Metallic	4		
5	Octanal	B	Lemon-like, Fresh	2	Fresh, Lemon-like	1	Unpleasent, Green	3	Lemon-like, Fresh	4	Green	3	Lemon-like, Fresh	3		
6	Limonene	B	Fresh	2	Fresh, Lemon-like	2	Fresh, Fruity, Lemon-like	3	Fresh, Fruity	3	Cinnamon-like, Lemon-like	2	Fresh, Lemon-like	2		
7	Benzeneacetaldehyde	B	Green, Roasted	2			Green, Floral	2	Green, Floral	4	Floral	3			Floral	2
8	Linalool	B	Floral	4	Sweet, Floral	2	Sweet, Floral	3	Sweet, Floral	4	Floral	4	Floral	4	Sweet	2
9	p-Mentha-1,8-dien-7-ol	B	Green, Fatty	2	Sweet, Floral	2			Green, Floral	3	Green, Floral	3	Floral	1	Sweet, Floral	2
10	Citronellal	A	Green, Fatty	2	Green, Wood	2	Green, Fresh	3	Green, Fresh	4	Fruity	1	Green, Wood	3		

11	Decanal	B	Floral	3	Sweet	2	Floral	3	Fresh, Spicy	3	Sweet	3	Fresh, Sweet	3		
12	Octanoic acid ethyl ester	A	Fatty	3	Green, Waxy	3							Green, Waxy	3	Green	3
13	2-(n-Propyl)-pyrazine	A	Green, Fresh	2					Lemon-like	2			Green, Lemon-like	1	Green, Fresh	1
14	4-(1-Methylethyl)-benzaldehyde	A	Green, Herbal	2	Fresh	2	Fresh, Herbal	3	Green, Herbal	3	Fresh	3	Fresh	4	Fresh, Citrus peel	2
15	3-Phenyl-2-propenal	B	Cinnamon-like	4	Cinnamon-like, Sweet, Wood	3	Fatty, Bitterness, wood	3	Fresh, Fruity, Sweet	4	Cinnamon-like, Sweet, Wood	3	Fresh, Sweet	4	Sweet, Wood, Cinnamon-like	2
16	Bornyl acetate	B	Fresh	3					Fresh, Wood	2			Fresh, Wood	2		
17	Neryl acetate	A	Cinnamon-like	2	Cinnamon-like, Floral	1			Floral, Fruity	2	Floral	2			Floral	2
18	Copaene	B	Cinnamon-like	3			Fruity-wine	3	Sweet, Floral	4	Sweet, Floral	2	Sweet, Floral	2	Sweet, Wood	2
19	Dodecanal	B	Floral	3	Green, Waxy	1	Sweet, Medical, Green,	2	Green, Waxy	4			Green, Waxy	3		
20	β-Caryophyllene	A	Citrus juicy	3			Floral, Wine	3	Floral, Sweet	3			Floral, Fruity	2		
21	β-Cubebene	B	Fruity	3	Floral	1			Floral, Sweet	3	Fruity, Citrus-like	2	Fruity, Citrus-like	3		

22	Aromandendrene	B	Fruity	3	Cinnamon-like, Wood, Sweet	3	Milk, Bitterness, Wood	3	Floral	3	Fresh	3	Cinnamon-like, Wood, Sweet	3	Wood, Sweet	2
23	β-Famesene	C	Sweet, wood	3	Sweet, wood	1	Floral	2	Floral	4	Medical, Wood, Sweet	1	Wood	4		
24	γ-Decalactone	C		4	Sweet, wood	2	Floral, Coconut-like	2	Fresh, Coconut-like	3	Floral	3	Floral	3		
25	trans-β-Ionone	B	Sweet, Floral	3	Sweet, wood	1	Sweet, Floral	4	Coconut-like	4	Floral, Sweet	2	Floral	2		
26	α-Muurolene	B	Coconut-like	2	Wood	1							Wood	2		
27	α-Calacorene	C	Floral	2									Floral	3	Floral	2

Note: [1] AI represents aroma intensity.

Table S4. The detailed performance of GC-O analysis of Bergamot Group samples.

No.	Compounds	Class	Assessor 1		Assessor 2		Assessor 3		Assessor 4		Assessor 5		Assessor 6		Assessor 7	
			**Aroma characteristics**	**AI[1]**	**Aroma characteristics**	**AI**	**Aroma characteristics**	**AI**	**Aroma characteristics**	**AI**	**Aroma characteristics**	**AI**	**Aroma characteristics**	**AI**	**Aroma characteristics**	**AI**
1	Hexanal	A	Fruity	1	Fresh, Wood	2	Fresh, Fruity	2	Fresh, Fruity	4	Plastic	1	Green	2	Fresh, Green	2
2	2-Hexenal	B	Green	1					Fresh, Fruity	2			Fruity, Lemon-like	1		
3	Heptanal	A	Fatty, Green, Unpleasant	3	Green, Fatty	1	Fatty, Green	3	Green, Fresh	3	Fresh	3	Green, Fresh	3	Fresh	2
4	β-Myrecene	A	Green, Metallic	2			Metallic, Green	2	Green		Green, Fresh	1	Green	2	Green	3
5	β-Pinene	A	Medical, Metallic	2	Mushroom-like	3	Green, Wood	3	Rust, Green, Wood	3	Green, Citrus-like	3	Green, Wood	3	Green	2
6	Nerol	B	Floral, Sweet	2			Green, Wood	2			Sweet	1	Floral	2	Floral	3
7	4,6,6-Trimethylbicyclo[3.1.1]hept-3-en-2-one	B	Fatty	1	Sweet, Floral	2	Floral, Fruity	3	Fruity	2	Sweet	2	Fruity	3		
8	Limonene	B	Mint, Fresh	1	Lemon-like, Fruity, Fresh	1	Fresh, Mint	3	Fresh, Fruity	3			Lemon-like, Fruity, Fresh	2		

9	1-Methyl-4-(1-methylethenyl)-benzene,	E	Unpleasant	3	Wood	2	Unpleasant, Medical	3	Coffee-like	4	Unpleasant	2	Unpleasant	3	Wood	2
10	Linalool	B	Floral	2	Floral	1	Floral	3	Floral	4	Sweet	3	Floral	4	Floral	1
11	(E,E)2,6-Dimethyl-2,4,6-octatriene	A	Green, Fresh	1			Fresh, Floral	2	Fruity, Floral	3			Fresh, Wood	3		
12	3,6-Dihydro-4-methyl-2-(2-methyl-1-propenyl)-2H-pyran	A	Green, Unpleasant	1	Green	1	Green, Fatty	2	Green, Fatty	2	Green	2	Plastic	4	Green	2
13	Benzyl acetate	C	Cinnamon-like, Sweet, Herbal	2			Fresh, Herbal	3	Mlik-like, Sweet	2	Herbal, Medical	2				
14	Terpinen-4-ol	A	Fresh, Mud	2	Fresh, Herbal	1	Medical	4	Fresh, Wood	4	Mint	2	Wood	4		
15	α-Terpineol	B	Fresh, Herbal	1	Floral	1	Green	4	Mlik-like, Floral	2	Fruity, Floral	2				
16	Linalyl acetate	B	Fresh, Citrus-like	3	Herbal	3	Floral	3	Fresh, Mint	3	Citrus-like, Herbal	2	Citrus-like	2	Fresh, Herbal	2
17	Isopulegol acetate	C	Sweet, Fruity	2	Wood	2			Sweet, Wood	3						
18	Geranyl acetate	B	Floral, Violet-like	3	Sweet	2	Floral	4	Sweet	3	Floral	1	Floral	4	Floral	3
19	Decanoic acid, ethyl ester	A	Green	1					Fatty	3			Green	3		

20	α-Ionone	B	Floral	1	Floral, Sweet	1	Mike-like, Sweet	3					Sweet	4		
21	Nerolidol	B	Fruity	2			Floral	3	Fruity	2			Fruity	2	Floral	2
22	trans-β-Ionone	B	Floral, Violet-like	2			Floral	3			Floral	4				2
23	Caryophyllene oxide	C	Sweet, Herbal	1	Herbal	2	Herbal	1								

Note: [1] AI represents aroma intensity.

Table S5. The detailed performance of GC-O analysis of Lemon Group samples.

No.	Compounds	Class	Assessor 1		Assessor 2		Assessor 3		Assessor 4		Assessor 5		Assessor 6		Assessor 7	
			**Aroma characteristics**	**AI[1]**	**Aroma characteristics**	**AI**	**Aroma characteristics**	**AI**	**Aroma characteristics**	**AI**	**Aroma characteristics**	**AI**	**Aroma characteristics**	**AI**	**Aroma characteristics**	**AI**
1	2-Ethylfuran	E	Unpleasant, Medical	1					Unpleasant, Roasted	3	Unpleasant, Medical	2			Unpleasant	
2	Hexanal	A	Fresh, Green	1	Fruity	1	Green, Fresh	2	Fruity	3	Green	3	Green, Fresh	2		
3	Furfural	D	Roasted	1			Medical, Roasted	3			Roasted	2	Lemon-like, Fresh	3		
4	Heptanal	A	Plastic	2	Fresh	2	Green, Fresh	3	Floral	4			Fresh, Citrus-like	2	Fresh, Green	1
5	Benzaldehyde	E	Medical	1	Unpleasant	1	Plastic	2	Unpleasant	4			Unpleasant, Medical	3		
6	β-Pinene	A	Plastic	3	Green, Wood	2	Sweet, Floral	3	Metallic	4	Green	3	Green, Wood	4		
7	Carveol	A	Lemon-like, Fresh	1			Fresh, Green	2	Fresh, Sweet	3	Plastic	2				
8	Lemonene	B	Fruity	1	Fruity, Lemon-like	1	Fresh, Sweet, Mint	3	Floral, Fruity	3			Fresh, Lemon-like	3		
9	Benzeneacetaldehyde	B	Fresh, Floral	2			Medical	2	Floral	3	Floral	2				
10	α-Pinene	B	Floral	3			Green	3	Floral	4	Fruity	3	Floral	4	Floral	2

11	γ-Terpinene	C	Mud, Green	2	Floral	2			Herbal	2	Green, Fatty	2	Fresh, Mint, Herbal	2	Herbal	2
12	Salicylic acid	D	Roasted	1	Roasted	1	Roasted	3			Roasted	2	Nutty	3	Rust	2
13	1-Methyl-4-(1-methylethenyl)-benzene	E	Unpleasant	2	Unpleasant	2	Charry-like	4	Wood	3	Unpleasant, Wood	2	Fatty	3		
14	Linalool	B	Floral, Fresh	3	Sweet	1	Sweet, Floral	3	Sweet, Fruity	4	Floral	4	Roasted, Fatty	4	Citrus peel-like	3
15	1-Ethenyl-4-methoxybenzene	C	Fatty	2	Wood	1							Sweet, Wood	3	Wood	2
16	p-Mentha-1,8-dien-7-ol	B	Green	2	Floral, Fresh	2	Green, Metallic	2	Floral, Wood	3			Floral	2		
17	Isopulegol	E	Medical	3	Medical, Unpleasent	3	Green, Wood	3	Roasted	3	Herbal	2	Plastic, Unpleasent	3	Unpleasant	2
18	3-(Methylthio)-nonanal	A	Mud	2	Mud	2	Wood, Plastic	3	Green, Wood	4	Green	3	Green	4		
19	Verbenol	A	Fresh, Wood	2					Fresh, Herbal	3	Fresh	1	Mud, Herbal	2		
20	α-Terpineol	B	Floral, Fresh	2	Fruity, Sweet	1	Sweet, Bitter	3	Green, Wood	3	Floral	2	Floral	3		
21	(Z)-3,7-Dimethyl-2,6-octadienal	B	Fruity, Fresh, Lemon-like	2	Floral	2	Floral, Sweet	3	Fresh, Fruity, Lemon-like	3	Herbal, Fresh	2	Lemon-like	3	Lemon-like	1

22	Geraniol	B	Lemon-like, Fresh	3	Fresh	2	Floral	2	Fresh, Lemon-like	4	Lemon-like	3	Floral	4	Lemon-like	2
23	(E)-3,7-Dimethyl-2,6-Octadienal	B	Green, Fatty	2	Cinnamon-like, Floral	2			Lemon-like, Fresh	4	Plastic	2			Lemon-like	2
24	Citral	B	Fatty, Green	2	Lemon-like	3	Medical	3	Lemon-like, Fresh	4	Medical	2	Lemon-like	2		
25	2,6,10,10-Tetramethyl-1-oxaspiro[4.5]dec-6-ene	C	Fatty	2	Herbal	1			Fresh, Fatty	3	Herbal	2	Citrus-like	2	Herbal	2
26	Neryl acetate	B	Floral	1	Sweet	2	Fresh, Mint	3	Fruity, Sweet	4	Fruity	3	Floral	3	Floral	2
27	Geranyl acetate	B	Floral, Fresh	3	Sweet, Fruity	2	Floral	4	Floral, Sweet	4	Sweet, Fruity	3	Fruity	4	Floral	3
28	Jasmine lactone	B	Lemon-like, Fresh	2	Fruity	2	Floral	2	Floral	2			Floral, Sweet	4		
29	trans-β-Ionone	B	Floral, Violet-like	3			Floral	4			Floral	2			Medical	2

Note: [1] AI represents aroma intensity.
